# Interferometric laser imaging for respiratory droplets sizing

**DOI:** 10.1007/s00348-023-03610-1

**Published:** 2023-03-30

**Authors:** Livia Grandoni, Loïc Méès, Nathalie Grosjean, Giovanni Leuzzi, Paolo Monti, Armando Pelliccioni, Pietro Salizzoni

**Affiliations:** 1 Department of Civil, Building and Environmental Engineering, Faculty of Civil and Industrial Engineering, University of Rome La Sapienza, Piazzale Aldo Moro, 5, 00185 Rome, Italy; 2 Laboratoire de Mécanique des Fluides et Acoustique, University of Lyon, CNRS UMR 5509 Ecole Centrale de Lyon, INSA Lyon, Université Claude Bernard, 36 Avenue Guy de Collongue, 69134 Écully, France; 3 Italian Workers’ Compensation Authority (INAIL), Department of Occupational and Environmental Medicine, Epidemiology and Hygiene, Monte Porzio Catone (Rome), Italy; 4 Univ Lyon, CNRS, Ecole Centrale de Lyon, INSA Lyon, Univ Claude Bernard Lyon 1, LMFA, UMR5509, 69130 Écully, France

## Abstract

Due to its importance in airborne disease transmission, especially because of the COVID-19 pandemic, much attention has recently been devoted by the scientific community to the analysis of dispersion of particle-laden air clouds ejected by humans during different respiratory activities. In spite of that, a lack of knowledge is still present particularly with regard to the velocity of the emitted particles, which could differ considerably from that of the air phase. The velocity of the particles is also expected to vary with their size. In this work, simultaneous measurements of size and velocity of particles emitted by humans while speaking have been performed by means of Interferometric Laser Imaging Droplet Sizing (ILIDS). This technique allowed us to detect emitted particles with size down to 2 µm as well as to quantify all three components of the velocity vector and the particle concentration. The outcomes of this work may be used as boundary conditions for numerical simulations of infected respiratory cloud transmission.

**Graphical abstract:**

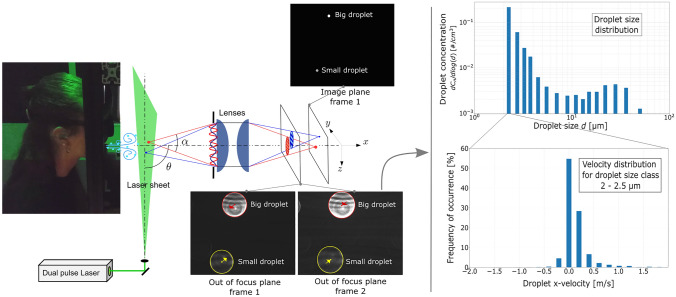

## Introduction

The particle-laden air cloud ejected by humans during different respiratory activities has drawn the attention of the scientific community since the first half of the twentieth century as it is involved in airborne disease transmission. This problem has become of major importance in the past 2 years due to the COVID-19 pandemic. Even though numerous experiments and numerical models have recently been carried out to assess the dynamics of dispersed cloud ejection (De Padova and Mossa 2021; Dbouk and Drikakis 2020a, b; Bourouiba et al. 2014; Busco et al. 2020; Wang et al. 2020; Chaudhuri et al. 2020; Li et al. 2022; Wei and Li 2015, 2017; Xie et al. 2007; Buonanno et al. 2020), a lack of knowledge regarding the way in which particles are ejected by humans is present yet (Rosti et al. 2020; Seminara et al. 2020; Mittal et al. 2020). Since model results often influence decision-making bodies in politics and public health, it is imperative for these dispersion models to be properly run with reliable initial data before their prediction may be used with a certain degree of confidence. In particular, experimental characterization of cloud ejection is useful to provide realistic data concerning air velocity as well as particle size and velocity distributions at the emission point. These quantities are important as ejected particle velocity and air velocity are expected to be different, particularly for the larger particles. Besides, particle velocity could vary with particle size. Particle size distribution and air velocity have been measured in several works, but only a few researches focused on the simultaneous measurements of particle size and velocity.

Size distribution of particles ejected during different respiratory activities, i.e., breathing, speaking, coughing, and sneezing has been analyzed, among others, by Han et al. (2013); Johnson et al. (2011); Asadi et al. (2019) (see also the review by Gralton et al. (2011) and the references cited therein). Different experimental techniques have been used in the past to measure particle size distribution, i.e., solid and liquid impaction (Duguid 1946; Loudon and Roberts 1967), optical particle counter (Han et al. 2013; Papineni and Rosenthal 1997), aerodynamic particle sizer (Asadi et al. 2019; Morawska et al. 2009; Johnson et al. 2011), electric low pressure impactor (Hersen et al. 2008), scanning mobility particle sizer (Holmgren et al. 2010), high-speed photography (de Silva et al. 2021) and Interferometric Laser Imaging for Droplet Sizing (ILIDS) (Chao et al. 2009). Despite the large availability of data, the works show considerable differences in the results (Bourouiba 2021; Johnson et al. 2011; Seminara et al. 2020; Mittal et al. 2020). This lack of agreement between different data sets enlightens the hurdles that have to be faced when characterizing experimentally this complex phenomenon.

The velocity of the ejected air has been measured by means of particle image velocimetry (Zhu et al. 2006; Chao et al. 2009; VanSciver et al. 2011), real-time shadowgraph imaging (Tang et al. 2013) and high-speed imaging (Nishimura et al. 2013). Alternatively, the airflow has been measured by means of spirometers (Mahajan et al. 1994; Singh et al. 1995; Gupta et al. 2009, 2010; de Silva et al. 2021). Only in a few cases experimental setups based on high-speed imaging techniques have been employed to measure the velocity of the ejected particles directly (Bahl et al. 2020; Nishimura et al. 2013; Scharfman et al. 2016; de Silva et al. 2021; Bahl et al. 2021). Even more rare are the simultaneous measures of particle size and velocity. This kind of measurement is not easy due to the small size of the particles and their low concentration. To our knowledge, only two studies, i.e., Wang et al. (2020) and de Silva et al. (2021), faced this problem, but only for large particles. In the former paper, the authors carried out a joint pdf of particle size and velocity for particles larger than 250 µm using particle shadow tracking velocimetry technique. de Silva et al. (2021) measured simultaneously particle velocity and size down to 36 µm using a back illumination and a high-speed camera. In both cases, only the vertical and the streamwise (normal-to-the-mouth) velocity components of the particles were measured, while no information about the spanwise component was available.

To provide an experimental characterization of the particles ejected by humans during different respiratory activities—namely measuring the three velocity components and size of the particles—we adopt here the ILIDS technique, which has so far never been used for this purpose. ILIDS is an interferometric technique based on a laser sheet illumination and an out-of-focus image recording. It was originally developed for liquid spray by Glover et al. (1995) based on a previous work of Ragucci et al. (1990). The basic idea is to combine the high accuracy of interferometric techniques with the capability of image analysis techniques to separate and identify several objects individually. The technique has been applied in several configurations (Dehaeck and van Beeck 2008; Porcheron et al. 2015; Rezaee and Kebriaee 2019; Sahu et al. 2016; Yilmaz et al. 2021) and extended to velocity measurements (Maeda et al. 2000) and bubble size measurements (Kawaguchi et al. 2002; Mees et al. 2011). One of the strengths of ILIDS is that it permits the simultaneous measurement of particle size and velocity using a Particle Image Velocimetry setup (double cavity laser and double frame camera or high repetition rate pulsed laser and camera). Another advantage of ILIDS is that it allows liquid droplets to be distinguished from suspended irregular solid particles even in a real environment, out of cleanroom conditions. Even though its applications are generally limited to diluted sprays, ILIDS is well suited to measure the low concentration of respiratory aerosols as well. A limitation of the ILIDS concerns the minimum size of the droplets that can be recognized, which depends on the aperture angle of the collection optic. In principle, a collection angle of about 40° is required to measure water droplet size down to 2 µm, which is far above the effective aperture of standard optics (note that contrary to the common usage in the literature concerning airborne disease transmission, the term *droplet* is used in the present paper to mean liquid particles in general, including small liquid particles). To apply ILIDS to respiratory droplets and recognize particles with very small diameter, a large aperture optic, free from spherical aberration, must be designed. Finally, the estimation of the measurement volume and the related particle concentration is not an easy task using ILIDS in that it is not possible to fix the size of the measurement volume a priori. For this reason, the measurement volume and its variation with the particle size have been estimated in the present work based on droplet location along the direction normal to the laser sheet.

The paper is organized as follows: Section 2 describes the principle of the standard ILIDS technique along with the improvements made to (i) detect droplets down to 2 µm, (ii) measure the three velocity components and size of the particles and (iii) determine the measurement volume for each size class and, hence, the droplet concentrations. Section 3 describes the experimental setup, the experimental protocol, and the image processing procedure. The results are presented in Sect. 4, enlightening the potential of the ILIDS technique for respiratory droplets characterization. Conclusions are summarized in Sect. 4.

## ILIDS technique

### Basic principles

**Fig. 1:**
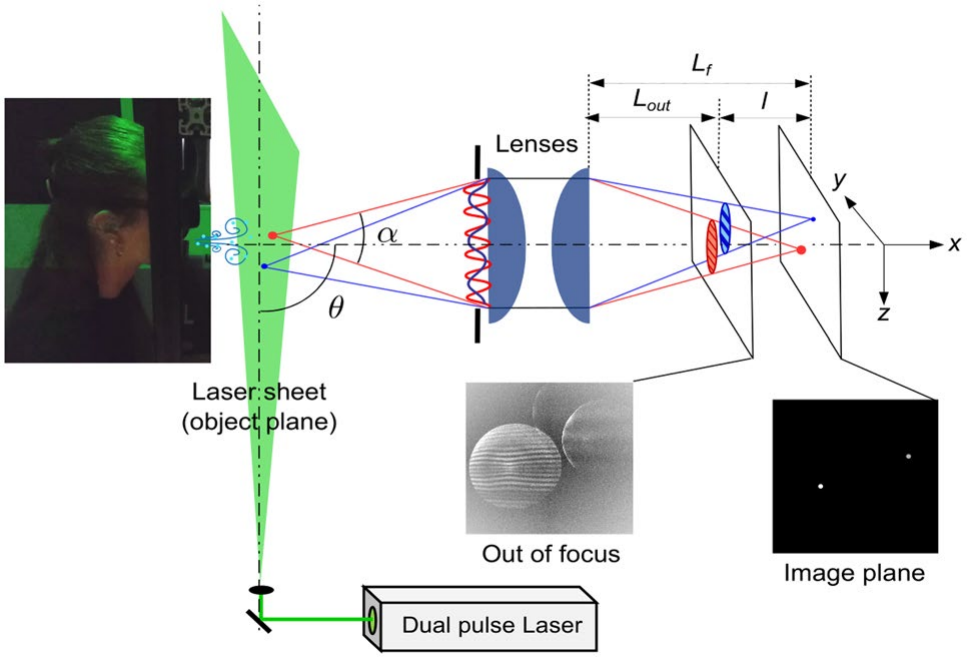
Sketch of ILIDS technique principle. A laser light sheet illuminates the particles. A portion of the light scattered by the particles is collected by an optical system (lenses) along the direction identified by $\theta$. The angle formed between the particle and the effective lens aperture is the collection angle $\alpha$. Out-of-focus images of the particles are taken by means of a camera located at a distance $L_{\text {out}}$ from the lenses. The light scattered by the particles is characterized by interference fringes, whose frequency is related to the particle size. Therefore, in the out-of-focus image, the particles appear as circles with interference fringes inside. Differently, in the image plane (at a distance $L_f$ from the lenses), the particles appear as glare points. The out-of-focus images become more and more blurry as the distance *l* increases

The ILIDS technique is based on the light scattering properties of transparent particles. As shown in Fig. 1, the droplets are illuminated by a coherent light source (the laser sheet) and the light scattered by the droplets is collected by a lens (or a lens assembly) with collection angle $\alpha$ centered around the direction identified by angle $\theta$. With a classical imaging system, the droplet images would form in the image plane at distance $L_f$. Conversely, with the ILIDS technique, the sensor is placed on an out-of-focus plane at distance $L_{\text {out}}$. The light scattered by a single droplet gives rise to an interference pattern (fringes) produced by the superposition of reflected light, refracted light and light refracted after one or multiple internal reflections. At a given angle $\theta$, the fringe spacing directly depends on the particle diameter. Incidentally, it is worth noting that while for a single drop, an accurate measurement of the drop size could be obtained by placing a sensor (instead of the lenses) at the lens location able to record the interference pattern (see Fig. 1), in the case of several droplets, the interference patterns would be superimposed on each other, and the information from individual droplets would be lost. With ILIDS, out-of-focus particle images are recorded by means of a lens (or a lens assembly) and a camera. In the out-of-focus plane, each droplet image takes the form of a circle containing the corresponding interference pattern. The circle location corresponds to the droplet location in the object plane (plane coinciding with the laser sheet where the observable objects, i.e., the particles, lay). Different droplets generate separated circles on the sensor, allowing the analysis of individual interference patterns and droplet size measurement. Note that, in ILIDS, the circle size is not related to the droplet size, but it only depends on the out-of-focus level, i.e., the ratio $l/L_f$. If the laser sheet was very thin, all the illuminated droplets would belong to the same plane, the image plane would be the same for all droplets, and all the corresponding circles would have the same size. For a thicker laser sheet, particularly when using a high magnification optic, both $L_f$ and circle size vary significantly with the distance of the droplet from the lens, and the third droplet coordinate can be deduced from the circle diameter. For a spherical droplet, the interference pattern is easy to detect and to count in that it is composed of regular fringes in a well-defined direction. Conversely, irregular solid particles can be easily recognized and discarded as they show less regular scattering patterns. For spherical droplets, the number of fringes in the circle equals the number of fringes that would be measured in the lens aperture, i.e., the number of fringes in the collected part of the scattering diagram. For scattering angles $20^{\circ } \le \theta \le 80^{\circ }$, the relation between fringe number and droplet diameter can be evaluated based on geometrical optics considerations. For $\theta =90^{\circ }$, as adopted in this work, the relation is deduced from Lorenz–Mie theory.

### Particle size measurement

**Fig. 2:**
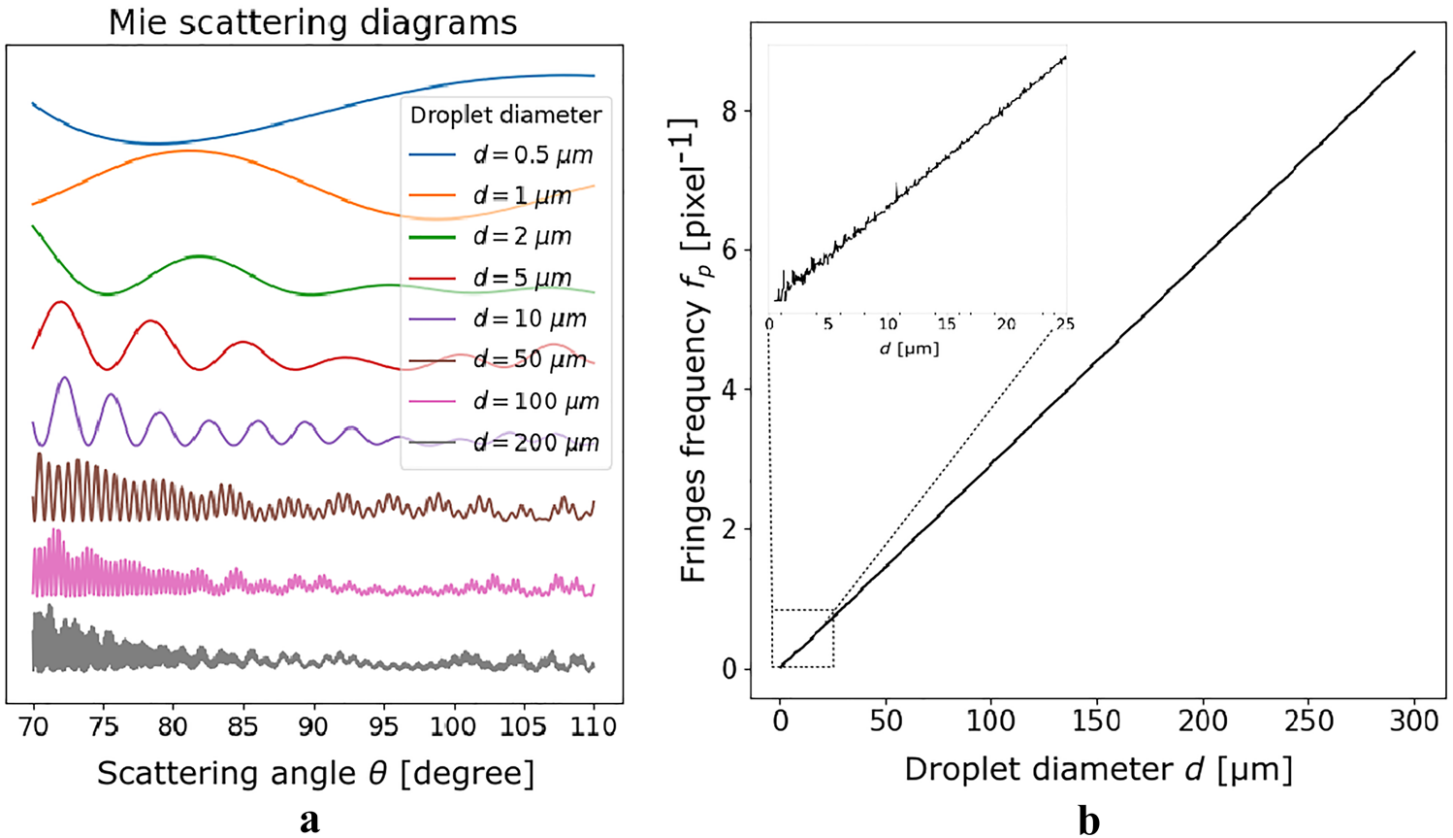
**a** Water droplet scattering diagram computed according to Lorenz–Mie Theory, for droplet diameters between 0.5 and 200 μm (from top to bottom of the left panel) and **b** Fringes angular frequency as a function of droplet diameter computed by using Lorenz–Mie Theory and Fourier analysis for scattering angle centered on $\theta =90^{\circ }$, a wide collection angle $\alpha = 40^{\circ }$, a wavelength $\lambda = 532$ nm and a sampling step on scattering angle $\delta _{\theta } = 0.05^{\circ }$

As mentioned in the former Section, the droplet diameter, *d*, can be determined in the scattering diagram from the angular spacing of the fringes, $\Delta \theta$. Assuming a perfect thin lens (i.e., of negligible thickness and not affected by any kind of aberrations) and a collection angle $\alpha$, $\Delta \theta$ can be written as follows:

1 $$\begin{aligned} \Delta \theta = \frac{\alpha }{N} = \frac{\alpha }{2 R f_p } \end{aligned}$$

where *N* and and *R* are the fringe number and the circle radius in out-of-focus image, respectively, while $f_p$ is the fringe frequency, measured in pixel unit. Figure 2a shows portions of the scattering diagrams for $70^{\circ } \le \theta \le 110^{\circ }$ ($\alpha = 40^{\circ }$ centered on $\theta = 90^{\circ }$) and droplet diameters lying in the range 0.5–200 µm. The laser beam is linearly polarized. The polarization direction has been chosen perpendicular to the plane of incidence (*x*, *z*) (S-polarization) to maximize the fringe contrast for the smallest droplets. Figure 2a also reveals that the number of fringes is too low to be measured for the smallest diameters, even with $\alpha =40^{\circ }$. The lower limit for droplet diameter measurement is then around 1.5 µm, corresponding to about 2 fringes in the collection angle. A smaller $\alpha$ would increase the lower limit, while an $\alpha$ significantly greater than $40^{\circ }$ can hardly be considered in practice for several reasons, as detailed below. Thus, $d_{\text {min}} = 1.5$ µm can be considered as the lower limit of the technique, and a wide collection angle (nearly 40°) is required to reach this limit.

The relation between the fringe spacing $\Delta \theta$ (or the fringe angular frequency ${\Delta \theta }^{-1}$) and droplet diameter can be established by using the Lorenz–Mie theory. Figure 2b depicts the main fringe frequency as a function of the droplet diameter for $\theta =90^{\circ }$, $\alpha = 40^{\circ }$, light wavelength $\lambda = 532$ nm and sampling step on scattering angle $\delta _{\theta } = 0.05^{\circ }$. This figure has been obtained by computing scattering diagrams similar to those presented in Fig. 2a for droplet diameters going from 0.5 to 300 µm and by extracting the main fringe frequency from the derivative of the Fourier transform of each diagram. As Fig. 2b also shows, the droplet diameter is proportional to the fringe angular frequency, viz.

2 $$\begin{aligned} d = \kappa \frac{1}{\Delta \theta } = \kappa \frac{2 R f_p}{\alpha } \end{aligned}$$

The coefficient $\kappa = 34.0 \mu m$ degree is calculated from a linear regression of the curve (Fig. 2b). This coefficient depends mainly on $\lambda$, $\theta$ and the droplet refractive index *n*. It also varies weakly with the collection angle ($\kappa =35.2 \mu m$ degree for $\alpha =10^{\circ }$ and $\kappa =33.9$ µm degree for $\alpha =44^{\circ }$).

For the smallest diameters (inset in Fig. 2b), oscillations due to both Mie scattering properties and signal sampling are observed. These oscillations clearly limit the accuracy of the measurement of the smallest particles, with an uncertainty greater than $40\%$ for diameters lower than 1.5 µm. This confirms that 1.5 µm is the lower particle diameter measurable with the ILIDS technique. To account for this unavoidable source of uncertainty, only droplets having* d*_min_ ≥ 2 μm will be considered in the remainder of this work. For * d*_min_ ≥ 2 μm, the absolute error is less than $0.45\mu m$.

The maximum measurable particle diameter $d_{\text {max}}$ depends on the sampling conditions. For the parameters adopted to compute the curve presented in Fig. 2b, in particular $\delta _{\theta } = 0.05$, the Nyquist frequency $f_{\text {max}}=10$ pixel$^{-1}$ corresponds to a maximum diameter * d*_max_ = 340 μm.

Note that the range of measurable diameters is also limited by the dynamic range of the camera sensor. The intensity of scattered light is roughly proportional to the particle diameter squared. Using a 16bit-camera with a low read noise, the diameter range is limited to one or two orders of magnitude.

**Fig. 3:**
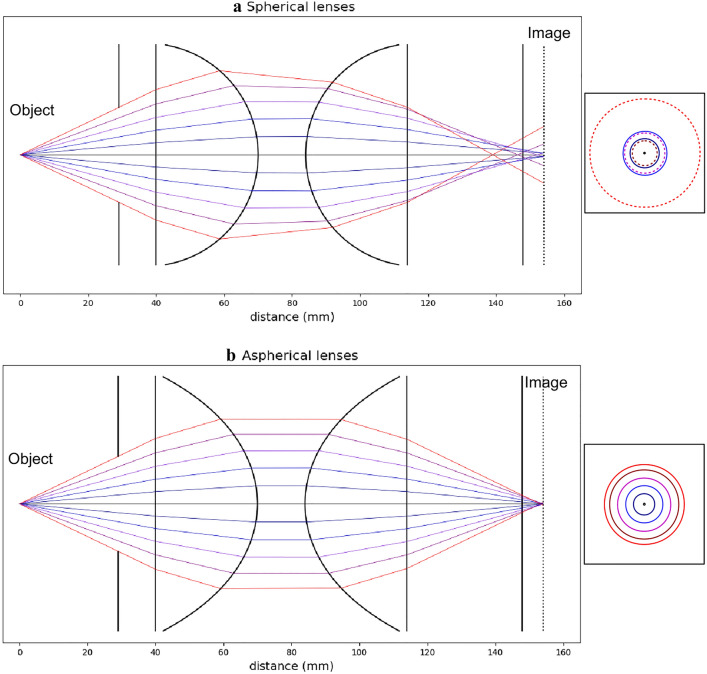
Effect of spherical aberrations on out-of-focus images. Using a pair of spherical lenses **a** the red rays at greater angles, far from the optical axis, cross this axis at shorter distances than the blue rays, closer to the optical axis. Using aspherical lenses **b** almost all the rays cross the optical axis at the same distance. As a consequence, a set of concentric circles corresponding to the different rays appears on the out-of-focus images. With aspherical lenses, the circle diameter increases linearly with ray angle. With spherical lenses, the circle diameter increases, then decreases and increases again, leading to a destructive folding of the fringes to be analyzed

To measure particle sizes down to 2 µm, the scattering diagram (Fig. 2a) must be collected over a large collection angle and entirely projected onto the sensor. In practice, the effective collection angle is limited by spherical aberrations as illustrated by the examples of ray tracing depicted in Fig. 3. When using a pair of spherical lenses (Fig. 3a), the rays collected at large angles (far from the optical axis) are focused at shorter distances. In the out-of-focus sensor plane, information carried by rays with different incidence angles overlaps and the projected interference pattern is folded in on itself and deformed, thus, preventing any measurement. The different ways to limit spherical aberrations are (i) to reduce the lens aperture, thus increasing the lower size limit, (ii) to increase the magnification ratio, reducing the field of view (hereinafter FOV) and the measurement volume, (iii) to increase the defocused level $l/L_f$ then increasing overlapping of defocused images and (iv) to use or design special lenses that reduce the spherical aberration. To reach an effective collection angle of order 40°, the last three solutions are applied together. Figure 3b illustrates the case of two aspherical lenses set in order to obtain a 1:1 magnification lens, nearly free from spherical aberration. In the out-of-focus plane (with the same out-of-focus level as in Fig. 3a), the different rays formed regularly spaced concentric circles, without any folding. This means that, despite the large collection angle (> 40°), the effects of spherical aberration are negligible. Note that this collection angle is however limited by an adjustable aperture placed in front of the first lens. To select the maximum aperture for which spherical aberrations remain negligible, the procedure consists in increasing it progressively while the circle diameter in the out-of-focus plane remains proportional to the aperture diameter. Preliminary to this work, several commercial lens assemblies have been tested. The largest collection angle has been obtained by using a 100 mm macro-lens (Zeiss Milvus 2/100 M) similar to the one used by Chao et al. (2009). The nominal aperture of this lens is 50 mm, but it must be reduced to less than 30 mm to fulfill the ILIDS requirements, leading to an effective collection angle of about 12° and minimum measurable diameter of about 6 µm. Note that this low performance of commercial lenses to compensate the spherical aberration is not surprising. Such lenses are optimized to reduce several kinds of geometrical and chromatic aberrations on focus images and not to reduce specifically the spherical aberration and its effect in a out-of-focus plane.

Even though aspherical lenses could be used to reduce spherical aberration, they are not perfectly thin and are prone to other geometrical aberrations. In particular, the image of an object away from the optical axis is deformed due to comatic aberrations. In the out-of-focus plane, the circles containing the interference patterns are deformed, in particular when the particle is close to the sensor edges. In the present case, the deformed circles are nearly elliptical and the effective collection angle is not affected significantly. The deformation does not prevent the measurement but it must be considered in the image processing phase. For droplets close to the top or the bottom of the image, the circle deformation is maximal in the direction perpendicular to the fringes, leading to a reduction in the fringe sampling condition of about one third. Therefore, the maximum measurable diameter for droplets in these image areas decreases by the same factor to about $d_{\text {max}} \sim 225$ µm.

### Particle velocity measurement

As mentioned earlier, the setup used in this work allows us to measure all three components of the particle velocity vector. These are determined from the particle coordinate changes between two images recorded with time delay $\Delta t$. The spanwise (*y*) and vertical (*z*) coordinates are evaluated from the circle center displacement in the sensor plane, while the streamwise coordinate (*x*, i.e., normal-to-the-mouth) is deduced from the circle size variation.

**Fig. 4:**
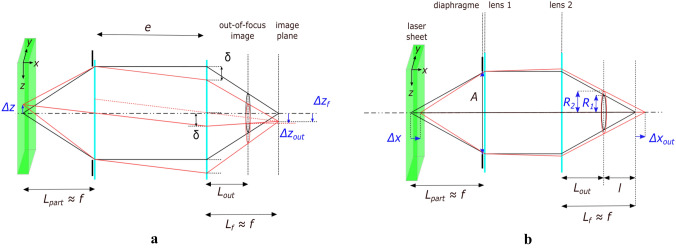
**a** *y*,*z*-displacement and **b** *x*-displacement. The conditions at two subsequent time steps are depicted in black and red. The displacements of the particles occurring between the two time step are $\Delta z$ and $\Delta x$. In case a, the displacement $\Delta z_{\text {out}}$ is observed in the out-of-focus image. In case b, the circle radius variation $\Delta R$ is observed in the out-of-focus image. The lines linking the particle in the laser sheet and its image are the central and the two extreme light ray paths

Let us consider a simple idealized lens assembly to illustrate how the circle position in the out-of-focus image varies with the particle displacement along the *z*-axis (or *y*-axis). The lens assembly is composed of two perfect thin lenses having the same focal length *f* and spaced at a distance *e* (Fig. 4a). For a particle located in the focal plane of the first lens, simple geometric considerations leads to the following relationship between the circle displacement, observed in the out-of-focus image $\Delta z_{\text {out}}$ (or $\Delta y_{\text {out}}$), and the real displacement of the particle $\Delta z$ (or $\Delta y$):

3 $$\begin{aligned} \Delta z_{\text {out}} = \Delta z \left[ \frac{e-L_{\text {out}}\left( \frac{e}{f}+1\right) }{f}\right] = -\gamma _1 \Delta z \end{aligned}$$

where $\gamma _1$ is a constant that depends on the optical system characteristics, i.e., *f*, *e*, the distance $L_{\text {out}}$ between the first lens and the out-of-focus sensor plane, and the aperture *A*.

As mentioned in Sect. 2.1, the radius of the circle associated with a particle depends on the distance between the particle and the optical system. As the laser sheet has a nonzero thickness, it is possible to detect the particle at different distances from the optical system (different positions within the laser sheet thickness). The third coordinate *x* can therefore be calculated from the circle radius *R*. Considering the same idealized optical system, with magnification $M\approx -1$, the circle radius for a particle located at $\Delta x$ from the focal plane of the first lens can be written as follows:

4 $$\begin{aligned} R(\Delta x) = \frac{A}{2}\left[ 1 + \frac{e \Delta x}{f (f-\Delta x)}\right] \left[ 1- \frac{L_{\text {out}}}{f-\Delta x}\right] \end{aligned}$$

The radius variation $\Delta R$, for $\Delta x$ close to zero, is nearly proportional to the displacement $\Delta x$, that is:

5 $$\begin{aligned} \Delta R \approx \frac{A}{2 f^3} \left( e f+ L_{\text {out}} f- e L_{\text {out}}\right) \Delta x = \gamma _2 \Delta x \end{aligned}$$

$\gamma _2$ is nearly constant for small displacement $\Delta x$ and depends on the optical system characteristics. Note that the variation of the circle radius *R* does not affect the measurement of droplet size *d*. Since $f_p$ varies inversely as *R* varies, the fringe angular frequency $1/\Delta \theta$ does not vary and so does the droplet size *d* (see Eq. 2).

To take into account for the real characteristics of the optical system used in the experiments and the inevitable default of optical adjustment, the two constants $\gamma _1$ and $\gamma _2$ have been estimated by means of a calibration procedure (see Sect. 3.2).

### Measurement volume and particle concentration

**Fig. 5:**
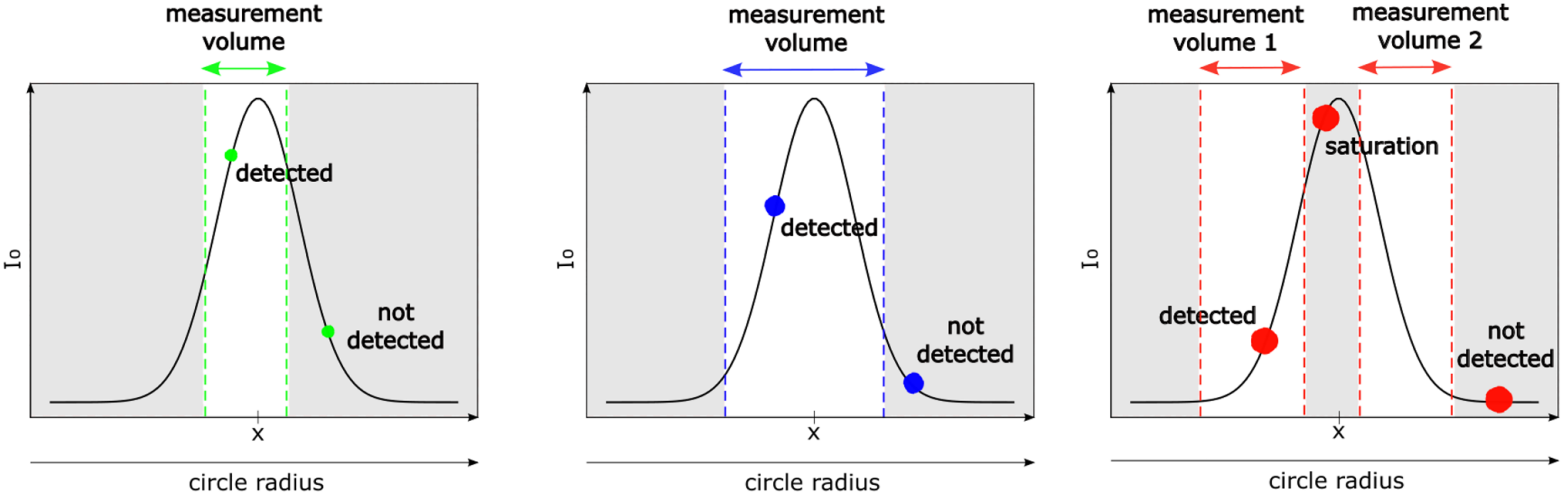
Detection range for particle of different sizes. Small, medium and large particles are represented in green (left panel), blue (central panel) and red (right panel), respectively. $I_{0}$ is the laser light intensity, while *x* is the position within the laser sheet thickness

ILIDS is based on a laser light sheet illumination that is supposed to delimit the measurement volume. However, the intensity of the laser sheet profile *I*(*x*) is never perfectly sharp, so that the identification of the laser sheet edges is not trivial. Moreover, dealing with a large size distribution, the effective measurement volume actually depends on the particle size (Fig. 5). Therefore, the measurement volume cannot be estimated a priori by multiplying the FOV with the thickness of the laser sheet. To be detected, the light intensity corresponding to a particle on the image must be greater than a given threshold that depends on the sensor sensitivity and noise level. Considering the real shape of the laser sheet profile, the actual width within which a particle can be detected decreases with its size. On the one hand, the intensity of the light scattered by a particle is roughly proportional to the square of its diameter and to the incident light intensity at the particle location. On the other hand, *I*(*x*) is not constant along the laser sheet thickness, but it is rather characterized by a smooth Gaussian-like shape. The measurement volume decreases with the particle size since smaller particles can be detected only at the center of the laser sheet, where the light intensity is sufficiently high. Conversely, the largest particles can be detected even in the edges of the laser sheet, where the light intensity is smaller, leading to a greater measurement volume. Note however that the largest particles located in the center of the laser sheet may lead to sensor saturation. This would prevent fringe frequency measurement, therefore leading to a measurement volume split into two parts. Besides, one should also consider the variation with *x* of the circle radius in the out-of-focus image. For smaller circle radii, the same amount of energy collected by the lenses is contained in a smaller area (circular surface in the out-of-focus image). Therefore, the minimum laser light intensity for which a particle of a given size can be detected is lower when the particle is positioned further away from the lenses, i.e., when the circle radius in the image is smaller.

The most reliable way to quantify the measurement volume for each particle size class is to deduce it *a posteriori* considering the actual *x*-coordinate variation observed for all the detected particles in a particle size class, *d*, through its standard deviation, $\sigma _x(d)$. This method has been previously used by Mees et al. (2011). The measurement volume for particle diameter *d* is then simply estimated as follows:

6 $$\begin{aligned} V(d) = \sigma _x(d) S \end{aligned}$$

where *S* is the FOV area.

## Experiments

### Setup and measurement campaign

To measure size and velocity of the particles ejected while speaking, twenty volunteers have been recruited to speak following the same protocol. To simulate the speech, they counted ten times from “one” to “one hundred.”

**Fig. 6:**
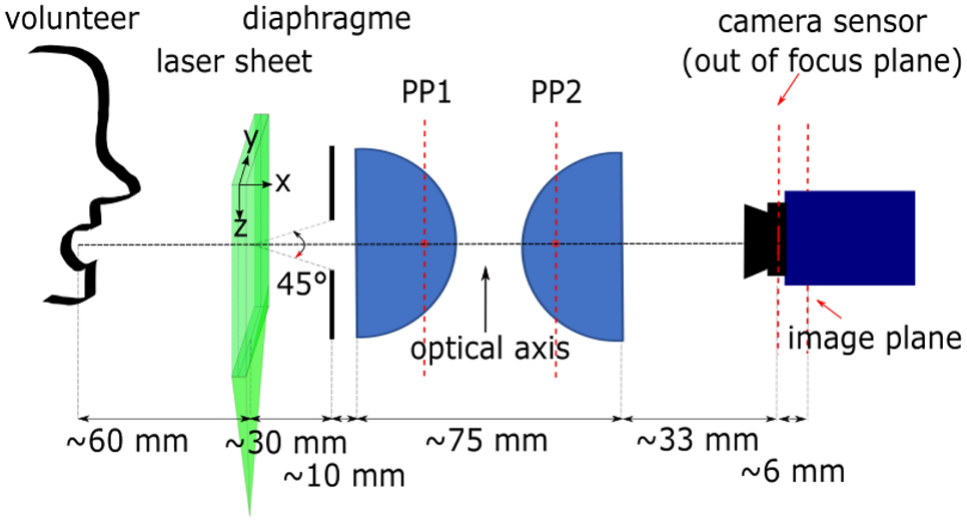
Schematic of the experimental setup. The principal planes (PP1 and PP2) of the two lenses are also drawn; these represent the position of the equivalent perfect thin lenses defined in Sect. 2.3

A double pulse Nd:YAG laser (Litron Bernoulli-PIV 200-15, wavelength: 532 nm, pulse duration: 8 ns, power: 2*200 mJ) was synchronized with a double frame camera (Lavision imager sCMOS, 16 bit, 2560 × 2160 pixel, pixel size: 6.5 × 6.5 µm, sensor size: 16.6 × 14 mm) by using a programmable timing unit. The acquisition frequency was set to 15 Hz (i.e., 15 couples of frames per second). The time delay between two consecutive frames, $$\Delta t$$, varied with the different respiratory activities, based on the expected range of particle velocity. On the one hand, $$\Delta t$$ must be high enough to observe a significant particle displacement. On the other hand, $$\Delta t$$ must be small enough not to let the particle exit the laser sheet. The optical system consisted of two aspherical lenses in series (Thorlabs ACL7560U, focal length: 60 mm, lenses aperture: 75 mm) and a diaphragm, which limited the effective lenses aperture to about 28 mm. The laser sheet was parallel to the lenses and to the mouth of the volunteer (see Fig. 6 for a schematic of the experimental setup). The location of the focus image plane was found experimentally. The corresponding magnification and field of view were $$M \approx \ 1$$ and FOV 14 × 17 mm^2^$$^{2}$$, respectively. The FOV was actually slightly narrower because of the cutting of a little portion of the images made in the image processing. Both the focus image plane position and magnification agreed well with those computed by a house made software calculating the light ray path derived from the properties of the optical system and laser sheet position. Given the distance between the laser sheet and the diaphragm, the collection angle was $$\alpha \approx \ 45^{\circ }$$—it slightly varied for particles located at the two edges of the laser sheet thickness.

A mask was used to allow volunteers to place their mouth close to the optical axis (Fig. 6). The mask protected also the face of the volunteers and prevented them from moving, therefore avoiding any contact with the laser sheet. Besides, a black paper panel was placed between the laser sheet and the volunteers’ body. The eyes of the volunteers were completely covered.

### Calibration

Several calibrations are required to consider the deformations due to comatic aberrations and to calculate the two coefficients $\gamma _{1}$ and $\gamma _{2}$ in Eqs. 3 and 5.

**Fig. 7:**
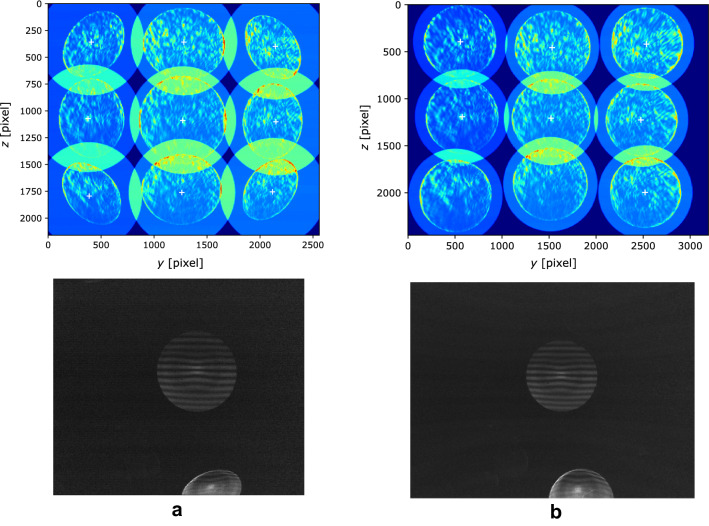
Upper panels: **a** superposition of 9 images of a point light source at 9 different distances from the optical axis and **b** the same images after deformation. Lower panels: example of image before (**a**) and after (**b**) deformation

As described in Sects. 2.2 and 3.1, two aspherical lenses in series are used to obtain an effective collection angle $\sim 45^{\circ }$, to measure particle size down to 2 µm. The deformation of the circles due to comatic aberrations not compensated by these lenses must be corrected by means of image pre-processing. This consists in applying a deformation to the images to retrieve a circular shape whatever the particle location in the image. To estimate the deformation to be applied, images of a point light source are taken at different distances from the optical axis (Fig. 7a, upper panel). The point light source consists of a pin-hole mounted on a 3D translation stage, illuminated at oblique incidence. A fifth-order polynomial deformation as a function of the distance from the optical axis is chosen. The result obtained by applying the deformation to the calibration images is shown in the upper panel of Fig. 7b. Note that this deformation is not physical, but it has the aim to make the automatic detection of the circles associated with the particles easier. Such deformation is necessary to simplify the detection of out-of-focus images, of different sizes and sometimes overlapping, by imposing a circular shape and thus reducing the number of free parameters for the detection. In the lower panels of Fig. 7, an example of image before and after deformation is depicted.

Similar images of a point light source at different positions are used to estimate $\gamma _{1}$. The ratio between the real displacement of the point light source and the displacement measured in the images is calculated. The calibration suggests that $\gamma _1$ is not perfectly constant over the whole FOV, with variations of ± 0.05 around a mean value $\gamma _{1} \sim \ 0.91$. Note that, considering an equivalent idealized optical system, as that presented in Sect. 2.3, with $f=60$ mm, $e=35$ mm, $L_{\text {out}}= 54$ mm and $A=56$ mm, Eq. 3 would lead to $\gamma _1 \sim 0.86$.

The proportionality between $\Delta R$ and $\Delta x$ (see Eq. 5) has been tested by displacing the point light source within the laser sheet thickness and measuring the circle radius. The coefficient $\gamma _{2}=0.38$ obtained from the experiments is comparable with $\gamma _{2}=0.44$, obtained for the idealized optical system from Eq. 5. The parameter $\gamma _{2}$ also varies slightly over the image, and its variations are also taken into account through the calibration.

### Image processing

The data consisted of $\sim \ 240,000$ images of about 15 MB each collected during the phase of speaking of the 20 volunteers. To deal with this large amount of images, a multicore version of the processing code, written in Python language, has been implemented on the high-performance computers available at the École Centrale de Lyon. To characterize droplet emission occurring over 2 min speaking (captured by 1200 images), data processing required about 96 h (CPU time). Using 32 cores on a HPC, the effective computing time was reduced to 3 h per test. Processing the data set acquired in the whole experimental campaign (240,000 images) would require 20,000 h CPU on a single core, which would be reduced to 600 h using 32 cores on HPC. In terms of memory, about 6 GB (Random Access Memory) per core are required. The image processing is composed of several steps, i.e., background subtraction, image deformation, particle detection, fringe analysis as well as velocity, size distribution and concentration calculation.

A background image is calculated for each test. It consists of the minimum intensity value for each pixel over all the images. The background image is subtracted to each image of the set.

Each image is deformed in order to compensate the aberrations as described in Sect. 3.2.

In the third step, all particles—both liquid and solid—are detected by means of a convolutive approach. A Gaussian filter is applied to filter out the interference patterns within the circles. The gradient of the image is then calculated, exhibiting the circle edges, and convoluted by synthetic ring images of different sizes to detect the circles and to measure their position and radius. The detected particles are removed from the image, and new particles are identified, until the correlation peak is above a fixed threshold. At the end of this step, radius and position of all detected particles are known.

In the fourth step, the interference pattern within the circles is analyzed and the fringe frequency is measured. When the circles overlap, only the non-overlapped part of them is analyzed. It is worth noting here that solid particles are easily recognizable (and therefore rejectable) in that they do not show regular horizontal fringes. This is a strong point of ILIDS technique applied to droplet recognition as it makes unnecessary to filter out ambient air to remove dust. The size of the liquid particles is then calculated by means of Eq. 2 (provided that $\alpha$ and *k* are known).

Particle size and position and circle radius are known at the end of the fourth step. Then, the three components of the particle velocity can be calculated by means of Eqs. 3 and 5 using $\gamma _1$ and $\gamma _2$ determined during the calibration procedure. The velocity components are directly inferred from associating the detected droplets in both frames once known the time delay $\Delta t$ between two consecutive frames. The association of droplets from both frames is made by minimizing both particle location and size changes between the frames.

Once repeated the four steps for all the images, size and velocity distributions can be built. The measurement volume for each particle size class, and therefore the particle concentration, can be estimated from Eq. 6.

## Results and discussion

In order to show the potential of the ILIDS technique employed in this paper, some preliminary results concerning the speaking activity are presented.

### Measurement volume

**Fig. 8:**
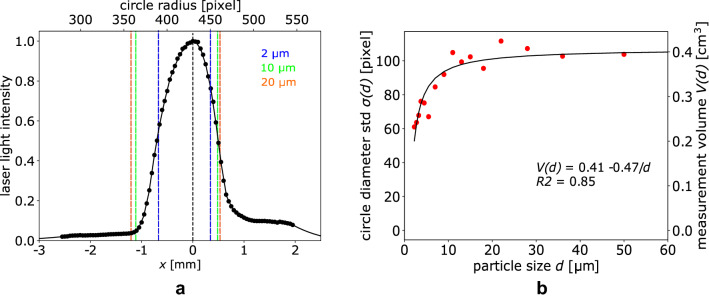
**a** Measured laser intensity profile along with detection limits for particles of 2, 10 and 20 µm, **b** Volume of measurement and related standard deviation of circle radius for each particle size

Figure 8b shows the measurement volume referred to a single frame for each particle size class along with the standard deviation of the circle radius, the latter proportional to the volume. The measurement volume increases almost linearly from 2 to 10 µm, while it remains nearly constant for $d > 20$ µm. The presence of such plateau can be ascribed to the weak variation of the light intensity at the laser sheet edges. However, the range of volume variation with the particle size is quite narrow, i.e., 0.2–0.4 cm^3^. As expected, the measured laser light intensity profile shows an almost Gaussian shape (Fig. 8a). The figure also shows that the ranges of *x* for which particles of 2, 10 and 20 µm have been detected. As mentioned earlier, smaller particles can be detected only at the center of the laser sheet, where the light intensity is higher. The circle radius corresponding to each *x*-position is also reported (see Eq. 5).

As already mentioned in Sect. 2.4, saturation is supposed to occur only for the larger particles (i.e., $d > 40$ µm). Nonetheless, saturation is not observed, probably because of a lack of data for the larger particles. Besides, since threshold estimation is based on light intensity measurement, it is intrinsically inaccurate.

### Size distribution

**Fig. 9:**
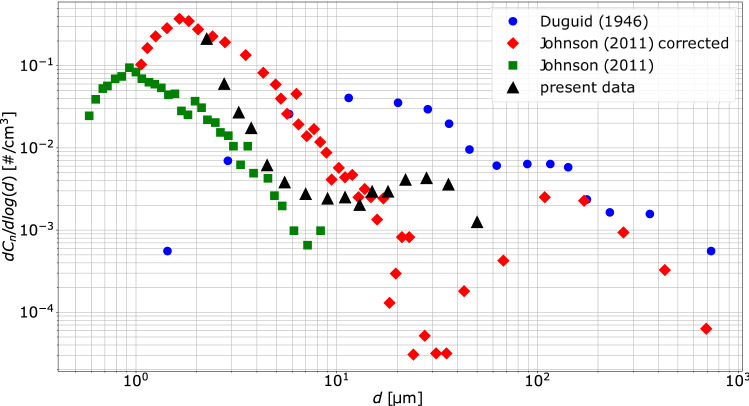
Measured particle size distribution, compared to data by Duguid (1946) and Johnson et al. (2011)

Figure 9 shows the particle size distribution obtained considering all the tests. For each size class, the particle number concentration normalized with the width of each bin size is given. The range of particle diameters detected during all the experiments is 2–60 µm, even though most of the particles lie in the size range 2–4 µm. The particle number decreases quickly up to $\sim 15$ µm. The absolute concentration maximum occurs for $d=2$ µm, while a local maximum takes place at $d\sim 28$ µm. The size distribution obtained with the technique presented here shows no droplet sizes greater than 60 µm (even though the upper limit of the technique is above 300 µm). This shows that these larger droplets are very rare in emissions occurring while speaking. Note however that the detection of these droplets may be affected by measurement errors that would excluded them from the statistics, in case, they contain solid or gas inclusions. Indeed, the presence of inclusions would lead to a much more complex fringes pattern. This would in turn result to a complex scattering pattern that would (erroneously) interpreted as representative of an irregular (solid) particle. For the interpretation of the complex patterns characterizing irregular particles, the reader is referred to the work by Brunel et al. (2014).

In Fig. 9, we also plot data from two other works found in the literature, i.e., the seminal work by Duguid (1946), which is considered as a main reference in the field, and that recently published by Johnson et al. (2011). In both the two works, the data were collected using almost the same protocol adopted here (for the respiratory activity associated with speaking). Johnson et al. (2011) detected a wide size range thanks to the use of a combination of two techniques, i.e., Aerodynamic Particle Sizer (APS) and Droplet Deposition Analysis (DDA). Note that APS data by Johnson et al. (2011) appear twice in Fig. 9. The green circles refer to APS data, while the red ones correspond to the same data set corrected for dilution and evaporation, together with the data provided by the DDA. Such correction had to be adopted since the sample probe of the APS was located far away from the volunteers’ mouth.

The size distribution obtained in the present work lies between the two Johnson’s distributions. We can explain this by recalling that, compared to Johnson et al. (2011), the measurement volume in our experiments is closer to the volunteers’ mouth and that we did not apply any correction to the data to take into account for evaporation and dilution effects. Our results also differ considerably from those by Johnson et al. (2011) for larger particles. Namely, we observe a concentration maximum at $d\sim 28$ µm in place of the minimum found by Johnson et al. (2011). However such minimum occurs for diameters in the range corresponding to that of the maximum diameter detectable by the APS and the minimum diameter for the DDA. Note also that the efficiency of the APS decreases as the particle size increases (Morawska et al. 2009). The size distribution of Duguid (1946) differs from that presented in this work and in Johnson et al. (2011). Nevertheless, Duguid (1946) stated that the size distribution he obtained should be taken with caution, because of the several approximations he made. For instance, Johnson et al. (2011) noted that the particle size estimated by Duguid (1946) could be shifted to larger sizes due to evaporation effect overestimation. Besides, since Duguid (1946) provided only the particle number, the particle concentration had to be calculated by estimating the air volume expired during the speaking activity (Johnson et al. 2011).

### Joint probability density function of particle velocity and size

**Fig. 10:**
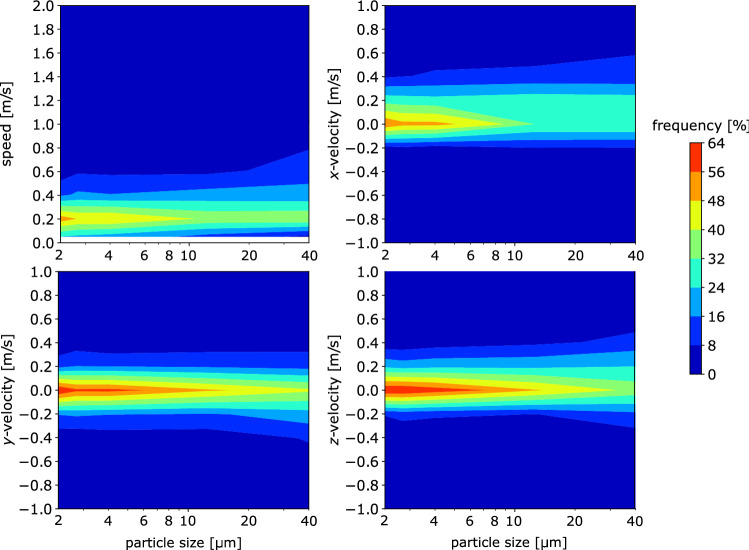
Joint probability density function of particle velocity and size. Colors are number of particles characterized by a certain size and velocity normalized by the total number of particles of each size

Figure 10 shows the joint probability density function (pdf) of particle velocity and size measured simultaneously in our experiments.

To build the joint pdf maps, five size classes have been considered in order to have robust statistics for each size class. All three velocity components depend on the particle size. In particular, the normal-to-the-mouth velocity component, $v_x$, grows with the particle size. On average, $v_x$ is about 0.1 ms$^{-1}$ for $2 \le d \le 2.5$ µm and 0.3 ms$^{-1}$ for $20 \le d \le 60$ µm. It is worthwhile noting the presence of negative $v_x$. These can be probably ascribed to air inspiration by the volunteer while speaking and particle recirculation due to vortices forming along the border of the air jet associated with the emitted air. As expected, the spanwise velocity component, $v_y$, has (nearly) zero mean value ($\sim \ 0.01$ ms$^{-1}$) due to flow symmetry for all the size classes. The vertical component of the velocity $v_z$ (positive downward) ranges between 0.02 ms and 0.05 ms^−1^ when $2 \le d \le 20$ µm, while it is higher ($\sim \ 0.1$ ms^−1^) for the largest particles, probably due to their settling.

Note that the theoretical minimum detectable value of the velocity components along *y* and *z* is $\sim \ 0.005$ ms^−1^, corresponding to a displacement of 0.5 pixel in 700 µs. The minimum velocity detectable along the *x*-direction is slightly higher, $\sim \ 0.02$ ms^−1^ (i.e., a variation of 1-pixel in the radius of the circle between two frames). The maximal detectable particle velocity is 7 ms^−1^. However, the maximum $v_x$ is limited by the thickness of the laser sheet where the particles can be effectively detected. Considering the values shown in Sect. 4.1, the maximum $v_x$ for 2 µm and 50 µm particles is 1.5 ms^−1^ and 2.5 ms^−1^, respectively. The maximal values of $v_x$ effectively measured are slightly higher. This is due to a slight underestimation of the *x*-particle detection range within the thickness of the laser sheet. The latter is, indeed, estimated by measuring the standard deviation of *x*, but some particles could be detected at an *x*-position which exceeds the $\sigma _d (x)$ considered.

The mean particle velocity observed in our experiments ranges between 0.26 and 0.56 ms^−1^ for the smallest and the largest particles, respectively. For comparison, the mean particle velocity measured by Wang et al. (2020); Bahl et al. (2021) and de Silva et al. (2021) is far higher. The larger particle sizes and the different respiratory activity—i.e., coughing and sneezing rather than speaking—can explain such difference with the current work.

### Particle direction

**Fig. 11:**
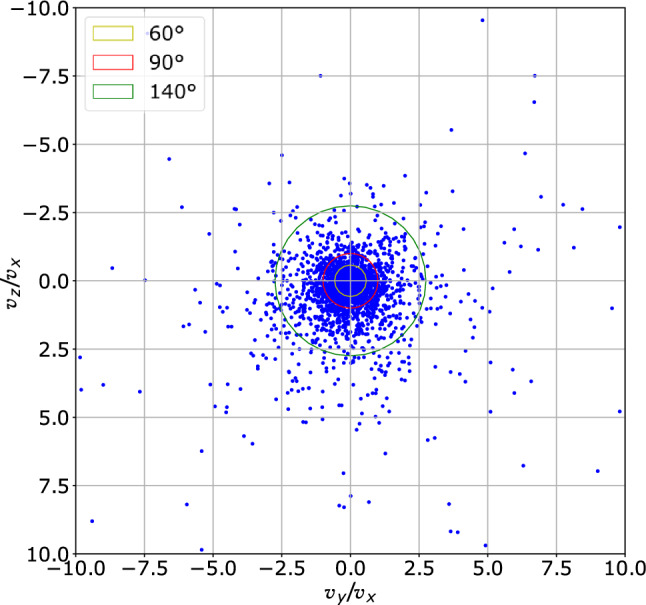
Ratios between the spanwise and the streamwise (normal-to-the-mouth) velocity components. The yellow (inner), red (intermediate) and green (outer) circles indicate an aperture angle of the particle cloud of 60°, 90° and 140°, respectively

Particle direction can be determined once the three velocity components are known. The ratios between the velocity components, $v_{z}/v_{x}$ and $v_{y}/v_{x}$, which are linked to the angle of departure of the particles from the *x*-axis (normal to volunteers’ mouth) are shown in Fig. 11. The ejected particle cloud appears symmetrical, and the aperture angle is quite wide. About 45–60% of the particles fall into an aperture angle of about 60° and 90°, respectively. Only a few particles depart from the mouth with an angle larger than 70°, corresponding to a ratio $\sim \ 2.5$. Note that only forward moving particles are considered in the figure.

### Uncertainty estimation

The droplet sizes are determined from the image circle radius and the fringes frequency measurements. The uncertainty on image radius is estimated to be 1-pixel. Given that the circles radius are of order 400 pixels, the relative uncertainty in droplet diameter is only 0.25%. The fringe frequency measurement relies on a sub-pixel determination. Considering an uncertainty of 0.5 pixel (probably overestimated), the resulting uncertainty depends on the fringe frequency itself and decreases linearly with the droplet diameter. For a 2 µm droplet, this relative uncertainty is about 15%, and it decreases to 1.5% for a 20 µm droplet. The droplet velocity in the x-direction is also deduced from the image circle radius in two consecutive images. Assuming a 1-pixel uncertainty on the image circle radius variation and given the 700 µs time lag between two frames, the relative uncertainty on the x-velocity is about 0.02 m/s (about 20% for a typical velocity of 0.2 m/s). Velocities in the y- and z-direction are determined from the displacement of the image circle center. These locations are determined by detecting a sub-pixel maximum in a convolution map. Assuming a 0.5 pixel uncertainty, the uncertainty on z- and y-velocity is of order 0.005 m/s (i.e., 10% for typical velocity of 0.05 m/s in those directions). Note that the uncertainties on velocity measurement can be easily reduced by a factor 2 or 4 by increasing the time lag between the frames by the same factor. Other factors that could potentially increase the measurement uncertainty are related to (i) the presence of droplet images not perfectly circular after image deformation, (ii) image circles partly cut at the edge of the image or (iii) background noise. These uncertainties are more difficult to assess individually. However, a global estimate of the droplet sizing uncertainty can be provided by the average difference between the droplet size measured in the two consecutive frames (as the same size should be measured for the same particle detected in the two frames). On average, the variation of the droplet size between the two frames, (normalized with the mean size) is about 4%; considering each size class individually, a maximum variation of 8% is observed for droplet size in the range 20–24 µm.

## Summary and conclusion

In this work, the Interferometric Laser Imaging Droplet Sizing (ILIDS) technique has been employed to determine size and velocity of particles emitted by humans while speaking. ILIDS made it possible to overcome some of the problems encountered in previous works, i.e., i. it is effective in measuring low particle concentrations, as in the case of humans’ emission; ii. it allows data collection close to the emission point and thus, it minimizes the effect of both dilution and evaporation; and iii solid irregular particles are easily recognized, so that the measures are not affected by dusts naturally present in the air (filtered-air chambers are not needed for the experiments).

Several improvements have been introduced over standard ILIDS in order to widen as much as possible the range of measurable diameters, in particular the smallest ones. In this way, particle diameters down to* d* = 2 µm and all three components of the particle velocity vector have been measured simultaneously. Besides, the variation of the measurement volume with particle size has been taken into account, making its estimation more reliable than that obtainable using more common measurement techniques based on light sheet illumination.

The main results that can be drawn from our experiments are:

1. The measurement volume increases linearly from 0.2 to 0.4 cm^3^ going from *d* = 2 to 10 µm; for bigger particles, it remains nearly constant.
2. The particle size distribution—expressed as particle number concentration—shows that most of the detected particles lie in the range 2–4 µm in diameter; a relative maximum of particle number concentration occurs at $d\sim 28$ µm.
3. The agreement between our data with the APS measurements carried out by Johnson et al. (2011) via Droplet Deposition Analysis is quite good for particle size between $2 \le d \le 10$ µm. In contrast, large differences with the results by Johnson et al. (2011) and by Duguid (1946) are observed for bigger particles.
4. The joint probability density functions of particle diameter and velocity components show a clear dependence of the normal-to-the-mouth velocity component on the size, e.g., $v_x$ ranges between 0.1 ms^−1^ for $2 \le d \le 2.5$ µm and 0.3 ms$^{-1}$ for $20 \le d \le 60$ µm.
5. The emission of the particles from the mouth is far from being unidirectional.

In spite of all its merits, the ILIDS technique has its drawbacks. For example, the size range of the ejected particles is not completely covered as particles smaller than 2 µm cannot be recognized. Some problems occur also for the larger particles, whose scattering diagram may differ from that expected theoretically due to possible inclusions of air (or other substances) or to their not perfectly spherical shape. Another limitation of ILIDS consists in the small field of view area needed to detect the smallest particles. Long measuring time is thus required in order to have robust statistics. The last aspect to be considered is that ILIDS does not work well in case of high droplet concentrations, which would lead to a more likely overlapping of their out-of-focus images. The overlapping of droplet (or irregular particles) images is considered by limiting the fringe analysis to the non-overlapping part of the images, but for images that overlap almost completely, the fringe analysis becomes impossible. In the case of speaking or breathing activities, the droplet concentrations are relatively low, so that droplet image overlapping occurs rarely and is easily managed. In case of coughing instead, the number of droplet images overlapping increases due to higher droplet concentration. Preliminary tests show that overlapping is still manageable, without changing the optical setup. In the case of sneezing, however, we estimate that the higher droplet concentration and the larger droplet sizes would require some adaptations of the setup, to reduce the laser sheet thickness, the out-of-focus level and possibly the collection angle. A development of the present work concerns the use of consolidated techniques and instruments such as FMPS (Fast Mobility Particle Sizer) and OPS (Optical Particle Sizer), which could be employed to analyze the size range (but not the velocity) of particles not detectable with ILIDS. A partial superposition of the measured size range could be useful also to compare the results. Furthermore, the reliability of the technique to characterize other respiratory activities is worth to be evaluated.
